# Degeneration of basal and limbic networks is a core feature of behavioural variant frontotemporal dementia

**DOI:** 10.1093/braincomms/fcab241

**Published:** 2021-10-21

**Authors:** Vesna Vuksanović, Roger T Staff, Suzannah Morson, Trevor Ahearn, Luc Bracoud, Alison D Murray, Peter Bentham, Christopher M Kipps, Charles R Harrington, Claude M Wischik

**Affiliations:** 1 Swansea University Medical School, Health Data Research UK, Swansea University, Swansea SA2 8PP, UK; 2 School of Medicine, Medical Sciences and Nutrition, University of Aberdeen, Aberdeen AB25 2ZD, UK; 3 TauRx Therapeutics, Aberdeen AB24 5RP, UK; 4 Medical Physics, NHS Grampian, Aberdeen AB25 2ZD, UK; 5 School of Psychology, University of Aberdeen, Aberdeen AB24 3FX, UK; 6 Bioclinica, Lyon 69003, France; 7 University Hospital Southampton and University of Southampton, Southampton SO16 6YD, UK

**Keywords:** behavioural variant frontotemporal dementia, brain networks, rich club, neurodegeneration, anatomical subtypes

## Abstract

The behavioural variant of frontotemporal dementia is a clinical syndrome characterized by changes in behaviour, cognition and functional ability. Although atrophy in frontal and temporal regions would appear to be a defining feature, neuroimaging studies have identified volumetric differences distributed across large parts of the cortex, giving rise to a classification into distinct neuroanatomical subtypes. Here, we extended these neuroimaging studies to examine how distributed patterns of cortical atrophy map onto brain network hubs. We used baseline structural magnetic resonance imaging data collected from 213 behavioural variant of frontotemporal dementia patients meeting consensus diagnostic criteria and having definite evidence of frontal and/or temporal lobe atrophy from a global clinical trial conducted in 70 sites in Canada, United States of America, Australia, Asia and Europe. These were compared with data from 244 healthy elderly subjects from a well-characterized cohort study. We have used statistical methods of hierarchical agglomerative clustering of 68 regional cortical and subcortical volumes (34 in each hemisphere) to determine the reproducibility of previously described neuroanatomical subtypes in a global study. We have also attempted to link the structural findings to clinical features defined systematically using well-validated clinical scales (Addenbrooke’s Cognitive Examination Revised, the Mini-Mental Status Examination, the Frontotemporal Dementia Rating Scale and the Functional Assessment Questionnaire) and subscales derived from them. Whilst we can confirm that the subtypes are robust, they have limited value in explaining the clinical heterogeneity of the syndrome. We have found that a common pattern of degeneration affecting a small number of subcortical, limbic and frontal nodes within highly connected networks (most previously identified as rich club members or functional binding nodes) is shared by all the anatomical subtypes. Degeneration in these core regions is correlated with cognitive and functional impairment, but less so with behavioural impairment. These findings suggest that degeneration in highly connected basal, limbic and frontal networks is a core feature of the behavioural variant of frontotemporal dementia phenotype irrespective of neuroanatomical and clinical heterogeneity, and may underly the impairment of integration in cognition, function and behaviour responsible for the loss of insight that characterizes the syndrome.

## Teaser

Using trial baseline MRI scans from 213 behavioural variant frontotemoporal dementia patients, Vuksanovic et al. report that the four known cortical atrophy subtypes all share degeneration in core basal, limbic and frontal network hubs that correlates with cognitive and functional impairment irrespective of subtype.

## Graphical Abstract

**Graphical Abstract:**
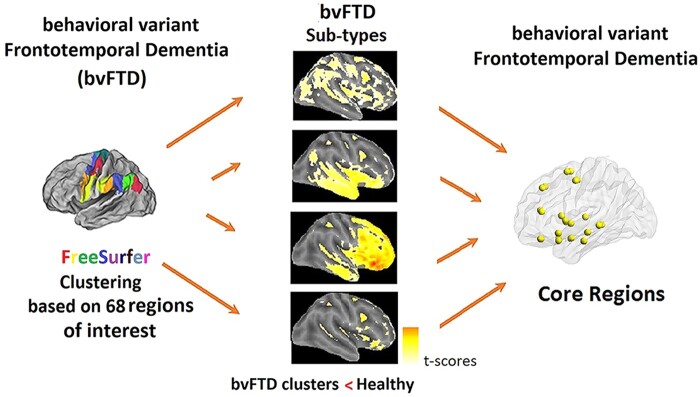

## Introduction

Frontotemporal dementia (FTD) is a heterogeneous disorder with distinct clinical phenotypes associated with multiple neuropathologic entities.^1^ The core FTD disorders are behavioural variant of frontotemporal dementia (bvFTD), semantic variant primary progressive aphasia and non-fluent variant primary progressive aphasia, but there are other disorders within the FTD spectrum that include FTD with motor neuron disease, progressive supranuclear palsy and corticobasal syndrome. Most cases of FTD have underlying tau, transactive response DNA-binding protein-43 (TDP-43) or fused in sarcoma, Ewing’s sarcoma or TATA-binding protein-associated factor 15 (collectively known as the FET protein family) neuropathology.^2^ bvFTD is a clinical syndrome characterized by insidious onset and progressive deterioration in behaviour, cognition and functional ability, the core symptoms being: disinhibition, apathy, lack of empathy, compulsions, hyperorality and impairment of executive function.^3^ Recently, higher levels of socially inappropriate behaviour and criminality in bvFTD compared with Alzheimer’s disease^4^ have been recognized, due to a dissociation between factual and evaluative understanding of actions and their consequences.^5^^,^^6^ Some patients may display the core clinical symptoms as a phenocopy syndrome that is not associated with brain atrophy.^7–9^ The revised diagnostic criteria for bvFTD therefore additionally require imaging evidence of a frontotemporal abnormality for a diagnosis of probable bvFTD.

Although atrophy in frontal and temporal regions would appear to be a defining feature, neuroimaging studies have identified volumetric differences distributed across large parts of the cortex, giving rise to a classification into distinct neuroanatomical subtypes.^10–14^ Distinct patterns of atrophy have also been associated with different types of FTD pathology and with mutations in different proteins.^15^^,^^16^ However, all pathological subgroups appear to share atrophy in the anterior cingulate, fronto-insula region, striatum and amygdala. Studies of genetic FTD have also shown that structural brain changes occur in insula at least 10 years before expected symptom onset.^17^ These vulnerable connected regions, which are affected early in bvFTD, are part of what has been termed the ‘salience’ network, which is thought to be responsible for processing of behaviourally salient stimuli in the normal brain.^15^^,^^18–20^ However, resting state network functional abnormalities may also extend to the default mode, frontoparietal and semantic appraisal networks resulting in other symptoms affecting attention, working memory and semantics.^11^^,^^21^ Recent evidence shows that the overlapping regions of resting state networks are organized into highly interconnected brain network hubs. Brain hubs are implicated in many types of dementia because of the role they play in integrating brain functions.^22^ Given the highly heterogeneous neuroanatomical subtypes in bvFTD, it is interesting to ask whether the anatomical heterogeneity involves these central brain regions, or whether atrophy in core regions is a common feature independent of cortical heterogeneity. We have previously reported the results of a voxel-based morphometry (VBM) comparison of baseline MRI scans of patients in a large randomized controlled clinical trial in bvFTD (TRx-237–007, NCT01626378) with those randomized to a comparable study of mild Alzheimer’s disease (TRx-237–005). This showed that the bvFTD group was clearly distinguishable from the mild Alzheimer’s disease group with a similar overall level of cognitive impairment.^23^^,^^24^ As expected, the bvFTD patients had significantly more atrophy in frontal cortex and anterior temporal cortex, and significantly less atrophy in hippocampus, middle temporal gyrus, cuneus and insula.

We undertook this study to determine how the apparent cortical heterogeneity of bvFTD subtypes relates to atrophy in central brain hubs and whether the subtypes are associated with distinctive clinical profiles. To address these questions, we have used baseline structural MRI data collected from 213 bvFTD patients meeting consensus diagnostic criteria^3^ and having definite evidence of frontal and/or temporal lobe atrophy^25^ and compared this with data from 244 healthy elderly subjects from a well-characterized cohort study.^26^ In addition, the availability of systematically collected clinical baseline scores using validated cognitive and functional scales provided the opportunity to determine how neuroanatomical subtypes and atrophy in brain networks relate to distinctive clinical profiles. Whilst, we confirm the existence of the subtypes, we show that they have limited ability to explain clinical heterogeneity in bvFTD. On the other hand, we report that the subtypes share a common pattern of degeneration affecting a small number of highly connected nodes, which have a subcortical, limbic and frontal distribution. Degeneration in these nodes is highly correlated with cognitive and functional impairment irrespective of subtype.

## Materials and methods

### Study participants

Study TRx-237–007 was designed as a 52-week Phase 3, randomized, controlled, double-blind, parallel-group trial conducted at 70 sites in Canada, USA, Australia, Asia and Europe. Eligible patients had to be younger than 80 years of age with a diagnosis of bvFTD according to criteria revised by the International bvFTD Criteria Consortium,^3^ with Mini-Mental Status Examination (MMSE)^27^ score ≥20 at screening. There was an additional requirement that patients had to meet the criterion of having definite brain atrophy in frontal and/or temporal lobes scoring two or more on a scale proposed by Kipps.^25^ Concomitant use of acetylcholinesterase inhibitors or memantine (or both) was permitted provided this was at a stable dose for at least 18 weeks before randomization. Concomitant use of serotonergic antidepressant, antipsychotic (except clozapine or olanzapine) and sedative medications was also permitted at stable doses where clinically feasible. Each patient had one or more study partners participate with them in the trial as informants. Patients were excluded from the study if they had a significant CNS disorder other than bvFTD. A detailed list of inclusion and exclusion criteria in the protocol is available in the Supplementary material in Shiells et al.^23^ As reported in that paper, the mean (SD) time since diagnosis of bvFTD was 1.9 (2.4) years, the MMSE severity at baseline was 24.6 (3.1) and 82.3% of the cases were at Kipps MRI severity stages 2 or 3. Of the 7/159 cases in whom mutations in coding regions were identified, 6 involved the *MAPT* gene for tau and one implicated the *TARDBP* gene for TDP-43. Baseline MRI scans were evaluated by a single independent neuroradiologist out of a pool of trained neuroradiologists to determine eligibility (RadMD, NY).

In addition, MRI scans were obtained from 244 age-matched healthy controls from the well-characterized Aberdeen 1936 Birth Cohort (ABC36) brain imaging database held in the Aberdeen Biomedical Imaging Centre at the University of Aberdeen. The ABC36 project has been described elsewhere.^26^^,^^28^ Demographics and clinical characteristics of the study groups are given in Table 1.

**Table 1:** Demographic and clinical data on bvFTD and healthy elderly subjects

Clinical Characteristics	bvFTD			
	All subjects mean (SD)	All subjects Range	Males mean (SD)	Females mean (SD)
	***N* = 213**		***N* = 136**	***N* = 77**
Age (years)	63 (7)	42-70	63 (7)	63 (7)
Education (years)	12 (4)	4–23	12 (3)	12 (4)
Handedness (R/L; A)	195/21; 2	(R/L; A)	123/13; 2	72/8; 0
eTIV (cm^3^)	1500 (220)	897–3077	1600 (220)^**^	1400 (150)
BF (TBV/eTIV)	0.67 (0.06)	0.37–0.94	0.67 (0.06)	0.68 (0.05)
MMSE (0–30)	24.5 (3.9)	11–30	25.4 (3.5)^**^	22.9 (4.0)
ACE-R (0–100)	68.5 (16.0)	17–99	72 (16)^**^	62 (14)
Attention (0–8)	3.9 (1.5)	0–8	4.4 (1.2)^**^	3.2 (1.7)
Orientation (0–10)	8.2 (1.9)	1–10	8.5 (1.8)^**^	7.7 (2.1)
Category fluency (0–7)	2.8 (1.9)	0–7	3.2 (2.0)^**^	1.8 (1.6)
Letter fluency (0–7)	2.8 (1.9)	0–7	3.1 (2.1)^**^	2.2 (1.7)
Language—phonemics (0–2)	1.5 (0.7)	0–2	1.6 (0.7)	1.4 (0.8)
Language—semantics (0–17)	12.7 (4.4)	1–28	13.2 (4.7)^**^	11.8 (3.8)
Language—structure (0–7)	6.2 (1.2)	2–11	6.2 (1.2)	6.0 (1.0)
Episodic memory (0–22)	12.8 (5.0)	0–22	13.3 (4.7)	12.1 (5.6)
Semantic memory (0–4)	2.4 (1.3)	0–4	2.6 (1.3)^**^	2.1 (1.2)
Perceptual abilities (0–8)	7.4 (1.2)	2–8	7.6 (0.9)	7.0 (1.5)
Praxis (0–8)	5.4 (2.3)	0–8	6.0 (2.0)^**^	2.4 (0.3)
FRS (−6.66 to 5.39)	−0.42 (1.45)	−3.80 to 5.39	−0.30 (1.4)	−0.62 (1.4)
ADL (fraction)	0.53 (0.25)	0–1	0.55 (0.26)^*^	0.48 (0.24)
Behavioural symptoms (fraction)	0.41 (0.22)	0–1	0.40 (0.22)	0.42 (0.23)
Cognition (fraction)	0.36 (0.38)	0–1	0.38 (0.40)	0.34 (0.37)
Apathy/disinterest (fraction)	0.38 (0.26)	0–1	0.39 (0.25)	0.37 (0.28)
Disinhibition (fraction)	0.39 (0.38)	0–1	0.35 (0.36)^*^	0.47 (0.39)
Eating behaviours (fraction)	0.60 (0.30)	0–1	0.60 (0.28)	0.63 (0.29)
Positive/problematic behaviours (fraction)	0.44 (0.27)	0–1	0.42 (0.27)	0.47 (0.27)
FAQ (0–30)	13.8 (7.2)	0–30	13.2 (7.1)	14.9 (7.2)
UPDRS (0–100) (%)	58 (11)	0–99	57 (10)	59 (13)
	**Healthy elderly**			
	**All subjects**	**All subjects** **Range**	**Males**	**Females**
	***N* = 244**		***N* = 133**	***N* = 111**
Age (years)	69 (2)	67–77	69 (2)	69 (2)
Education (years)	11 (2)	9–19	11 (2)	11 (2)
MMSE (0–30)	28.9 (1.2)	26–30	28.9 (1.1)	28.9 (1.2)

Significant between males and females:

* *P<*0.05,

** *P<*0.01.

R = right; L = left; A = ambidextrous; eTIV = estimated Total Intracranial Volume; TBV = Total Brain Volume; BF = Brain Fraction; ACE-R = Addenbrooke’s Cognitive Examination Revised; MMSE = Mini-Mental State Examination; FRS = Frontotemporal Dementia Rating Scale; ADL = activities of daily living; FAQ = Functional Assessment Questionnaire; UPDRS = Unified Parkinson’s Disease Rating Scale.

### MRI data collection

The acquisition protocol was standardized across sites using 1.5 T and 3 T scanners manufactured by General Electric, Philips or Siemens. All data were centrally collected, quality-controlled and analysed by the imaging core laboratory (Bioclinica). MRI data acquisition included a 3D sagittal T_1_-weighted sequence, which we used in our analysis here. 3D-T1 images were acquired using a 3D MPRAGE sequence (Siemens) or the specific manufacturer equivalent sequence (General Electric 3D IR-prepped Fast SPGR, Philips 3D TFE), covering the whole brain with a resolution of (1.25 **×** 1.25 **×** 1.2) mm^3^.

FreeSurfer version 5.3.0 (http://freesurfer.net/) was used to extract regional volumes for the clustering analysis. FreeSurfer automated segmentation parcelates the brain into 76 regions according to the Desikan–Killiany Atlas.^29^ For the purpose of this study, we selected 68 regional volumes (34 from each hemisphere) of the frontal, temporal or parietal lobe and additional sub-lobar (SL) regions (limbic lobes, basal ganglia, amygdala and thalamus) previously identified as locations of atrophy in bvFTD and/or as anatomical correlates of clinical symptoms. A full list of regions is given in the Supplementary Table 1.

Hierarchical agglomerative clustering, implemented in SPSS v.23.0, was used to classify differences/similarities in the 68 regional volumes. The bottom-up hierarchical agglomerative clustering is based on similarities and linkages between data points [subject-wise region of interest (ROI) volumes on MRI], with successive agglomeration of pairs of clusters until all clusters are merged into a single cluster containing all subjects. Similarity was measured by Euclidean distance between pairs of data points and linkage was measured by Ward’s linkage method.^30^ It is possible to classify bvFTD groups into either three or four clusters depending on the cluster distance. We used the 4-group clustering in further analyses for consistency with previous studies.^11^^,^^13^ Each of these groups was compared to the healthy elderly subject group using VBM.

**Figure 1:**
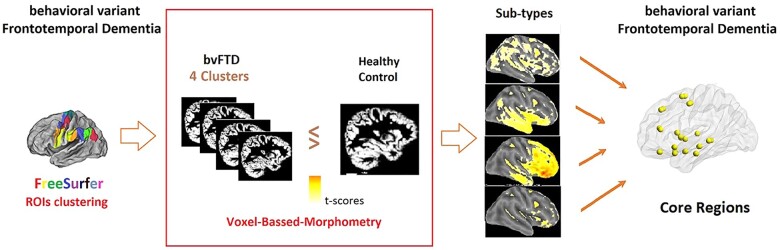
**Analysis pipeline.** A schematic of the analysis pipeline including Free Surfer ROI analysis, hierarchical clustering and VBM. Note the involvement of two study groups, healthy control and bvFTD, in different steps. Classification of bvFTD subjects into four clusters was done on 68 cortical ROIs extracted using Free Surfer. Each cluster was then compared with the healthy control group using VBM (red box) and anatomical bvFTD subtypes were identified from this analysis based on cortical atrophy (yellow areas on the cortical surface). The core regions are common to all four subtypes.

The VBM processing procedure employed for this study followed the steps described by Ashburner.^31^ In short, the images were first segmented into grey matter, white matter and cerebrospinal fluid mask images.^31^^,^^32^ Each class of the segmented images were then warped together and non-linearly registered so that they matched each other.^33^ A custom template was created from a data set of all participants in the study. Finally, images were normalized to the Montreal Neurological Institute (MNI) space and smoothed with a Gaussian kernel (8 mm FWHM). Each group identified by the clustering technique was compared to the healthy control group. Regions showing atrophy in the bvFTD group were identified from the MNI coordinates of the voxels within the areas that were significantly different using maximum difference *t*-test statistics. All image processing steps and statistical analysis were implemented in the Statistical Parametric Mapping (SPM12) software package available at http://www.fil.ion.ucl.ac.uk/spm/. The *t*-tests were performed on each pair of voxels/volumes corrected for age, gender and either estimated total intracranial volume (eTIV)^13^ or total brain volume^34^ to correct for global atrophy/severity. Recording sites were included in the model as a random covariate. To correct for the false discoveries of significant differences due to multiple tests, the *t*-test statistics were corrected at the significance level *P<* 0.05 using the family-wise-error correction available in the VBM statistical package. Figure 1 shows the overall procedure: generation of 68 ROIs in Free Surfer, classification of bvFTD subjects into four clusters using hierarchical clustering and cluster-wise comparisons with healthy control using VBM to determine regions affected by neurodegeneration.

### Clinical assessments

Baseline clinical assessments included the Addenbrooke’s Cognitive Examination Revised (ACE-R),^35^ the Mini-Mental State Examination,^27^ the Frontotemporal Dementia Rating Scale (FRS)^36^ and the Functional Assessment Questionnaire (FAQ).^37^ The bvFTD subtypes were compared using these scales and using subscales derived from the ACE-R and FRS prior to identification of the bvFTD subtypes. A total of 18 subscales were created: 11 cognitive subscales (from the ACE-R) and 7 behavioural subscales (from the FRS). The FAQ scale was used in its entirety as an independent measure of activities of daily living (ADL). The primary scales and derived subscales are described in greater detail in the Supplementary material.

### Statistical analyses

Statistical analyses were performed using SPSS v.23.0, employing paired samples *t*-tests to compare males and females in Table 1. One-way ANOVA was used to test differences between bvFTD subtypes in cognitive and behavioural sub-scores given in Table 1. A significance level of *P<* 0.05 was used.

In order to generate a summary variable accounting for the multiple regional volumes affected by neurodegeneration, we employed a *post-hoc* principal component analysis to reduce the volume measurements into a manageable number of factors. We examined clinical-anatomical correlations using general linear models (GLMs). In these we tested for associations between clinical measures and the first volumetric summary variable accounting for 40% of the variance. The models used included adjustments for known and potential confounders: age, sex, head size and anatomical subtype.

### Data availability

Data supporting the findings of this study are available from the corresponding author, upon reasonable request.

## Results

### Demographic and clinical features of the populations studied

A total of 213 bvFTD patients of 220 randomized to the trial were included in this study based on baseline MRI scan quality and the complete clinical data required for this study at baseline. Baseline demographic and clinical data are provided in Table 1. Mean (**±**SD) age was (63 **±** 7) years for both males (*N* = 136) and females (*N* = 77). Total years in education were 15.4 **±** 0.5, with no difference between males and females. The eTIV was significantly larger in males (1600 **±** 220) cm^3^ than in females (1400 **±** 150) cm^3^, although there was no difference in brain parenchymal fraction (0.67 **±** 0.06) cm^3^. The MMSE score was significantly higher in males (25.4 **±** 3.5) than in females (22.9 **±** 4.0), as was the total ACE-R score (males: 72 **±** 16; females: 62 **±** 14). Males performed better on most of the ACE-R subscales apart from phonemics, language structure, episodic memory and perceptual abilities. By contrast, there was no overall difference on either the FRS or the FAQ score between males and females. The only FRS subscales showing a gender difference were ADL (where males performed better) and disinhibition (where males performed worse). There were no demographic differences between patients prescribed symptomatic treatments approved for Alzheimer’s disease (but not bvFTD) and those not receiving these treatments.

There were 133 males and 111 females in the healthy elderly group. There were no sex differences in age, years of education or MMSE score. The healthy elderly group was significantly older (69 **±** 2) years, had less education (11 **±** 2) years, and had a higher MMSE score (28.9 **±** 1.2) than the bvFTD group (24.5 ± 3.9).

### Classification of bvFTD subjects by agglomerative clustering based on regional brain volumes

**Figure 2:**
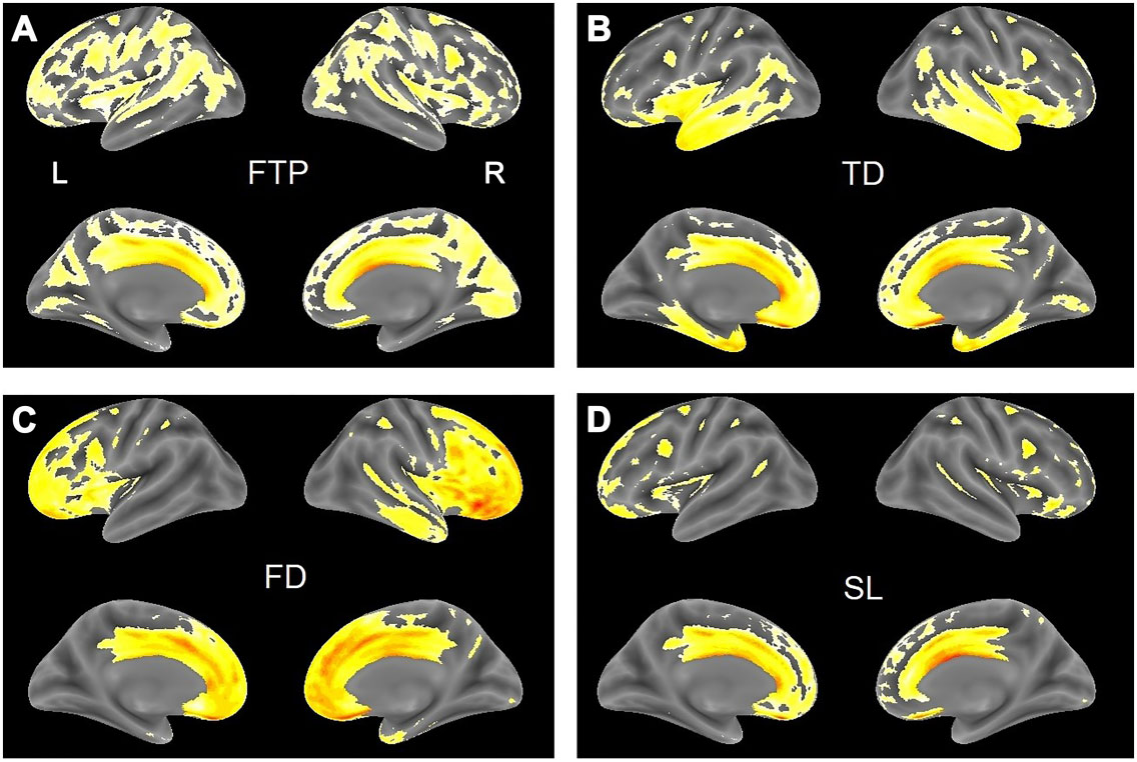
**Surface maps for four subtypes.** (**A**) FTP; (**B**) TD; (**C**) FD; and (**D**) SL. bvFTD individuals clustered based on differences in the 68 regional volumes. Yellow areas represent significant volume loss in each bvFTD cluster/subtype (sagittal and medial views) based on pair-wise comparisons with the healthy control group.

**Figure 3:**
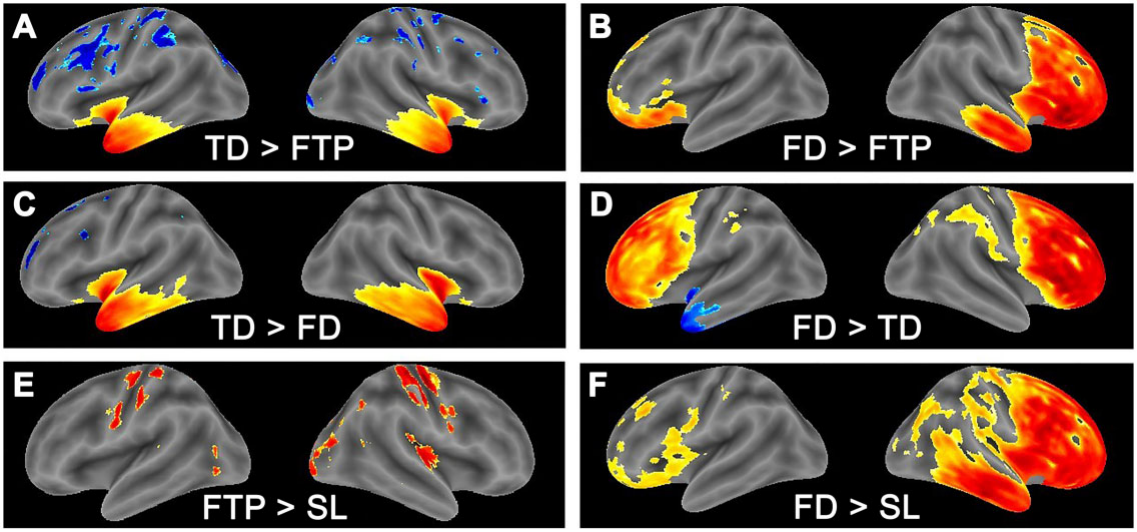
**Surface maps for differences between subtypes.** (**A**) TDP>FTP; (**B**) FD>FTP; (**C**) TD>FD; (**D**) FD>TD; (**E**) FTP>SL; and (**F**) FD>SL. Pair-wise differences between the four identified bvFTD clusters/groups mapped onto the cortical surface. Hot/cold colours indicate *t*-test statistics used for the voxel-wise comparisons. Hot colours indicate ‘more’ atrophy (as indicated in each panel by an inequality sign).

We applied a hierarchical agglomerative clustering algorithm using Euclidean distance and Ward linkage to provide measures of differences/similarities in the 68 regional volumes of the Desikan–Killiany Atlas.^29^ The tree/dendrogram is shown in Supplementary Fig. 1. It is possible to classify bvFTD groups into either three or four clusters depending on the cluster distance. We used the 4-group clustering in further analyses for consistency with previous studies.^11^^,^^13^ Each of these groups was then treated as a single group and compared to the healthy elderly subject group using VBM. Figure 2 shows the 3D surface rendering of the voxel-wise differences between each of the bvFTD groups and the healthy elderly group after correction for total intracranial volume. A similar result was found when the correction was based on estimated brain volume (Supplementary Fig. 3). Following Whitwell et al.,^14^ we designated the four anatomical subtypes: frontotemporoparietal (FTP), frontal-dominant (FD), temporal-dominant (TD) and SL. The differences in cortical atrophy across bvFTD subtypes are shown in Fig. 3.

### Degeneration of central basal and limbic nodes as a core feature of bvFTD

Having confirmed the classification of the bvFTD subtypes based on distinct patterns of cortical atrophy, we determined how these are linked to atrophy in subcortical and limbic regions (Supplementary Fig. 3 and Table 2). As shown in Tables 2 and 3, we found that it is possible to distinguish regions that are common to the four subtypes from those that are not. The limbic structures found to be common to all subtypes in terms of atrophy included anterior cingulate gyrus, hippocampal and parahippocampal gyri, insula and temporal pole (superior temporal gyrus). The subcortical grey nuclei affected in all subtypes included amygdala, caudate nucleus, pallidum and thalamus. The cortical regions common to the subtypes were the orbital surface areas of the frontal cortex (inferior frontal gyrus, olfactory cortex and gyrus rectus) and the medial surface areas of the frontal cortex (superior frontal gyrus and supplementary motor area). Only the middle temporal gyrus of the temporal lobe was shared between the subtypes. In all cases, the involvement of the common regions was bilateral.

**Table 2.** Cortical regions with differences in the brain matter volume common to all four identified bvFTD subtypes

Region	FTP (*N* = 82)	FD (*N* = 41)	SL (*N* = 39)	TD (*N* = 51)
Limbic lobe				
Insula	_**●**_	_**●**_	_**●**_	_**●**_
Cingulate_Ant	_**●**_	_**●**_	_**●**_	_**●**_
Hippocampus	_**●**_	_**●**_	_**●**_	_**●**_
ParaHippocampal	_**●**_	_**●**_	_**●**_	_**●**_
Temporal_Pole_Sup	_**●**_	_**●**_	_**●**_	_**●**_
Subcortical grey nuclei				
Amygdala	_**●**_	_**●**_	_**●**_	_**●**_
Caudate	_**●**_	_**●**_	_**●**_	_**●**_
Pallidum	_**●**_	_**●**_	_**●**_	_**●**_
Thalamus	_**●**_	_**●**_	_**●**_	_**●**_
Central region				
Precentral	_**●**_	_**●**_	_**●**_	_**●**_
Frontal lobe				
Frontal_Inf_Orb	_**●**_	_**●**_	_**●**_	_**●**_
Supp_Motor_Area	_**●**_	_**●**_	_**●**_	_**●**_
Olfactory	_**●**_	_**●**_	_**●**_	_**●**_
Frontal_Sup_Medial	_**●**_	_**●**_	_**●**_	_**●**_
Rectus	_**●**_	_**●**_	_**●**_	_**●**_
Temporal lobe				
Temporal_Mid	_**●**_	_**●**_	_**●**_	_**●**_

The group-wise differences at the whole-brain level were classified by VBM against the healthy elderly group using maximum of the *t*-tests statistics (separated by more than 1 mm) within a cluster and then labelled according to automated anatomical labelling implemented in SPM.

**Table 3:** Cortical regions with differences in the brain matter volume specific to either of four identified bvFTD subtypes (black-filled circles)

Region	FD (*N* = 41)	SL (*N* = 39)	TD (*N* = 51)	FTP (*N* = 82)
Limbic lobe				
Temporal Pole Mid L	_**○**_	_**○**_	_**○**_	_**●**_
Subcortical grey nuclei				
Putamen	_**○**_	_**●**_	_**●**_	_**●**_
Central region				
Rolandic Oper	_**●**_	_**○**_	_**○**_	_**○**_
Postcentral	_**○**_	_**●**_	_**○**_	_**●**_
Frontal lobe				
Frontal Sup	_**●**_	_**●**_	_**●**_	_**○**_
Frontal Mid	_**●**_	_**●**_	_**●**_	_**○**_
Frontal Inf Oper	_**●**_	_**●**_	_**●**_	_**○**_
Frontal Inf Tri	_**●**_	_**●**_	_**●**_	_**○**_
Frontal Med Orb	_**○**_	_**●**_	_**○**_	_**○**_
OFCant	_**○**_	_**●**_	_**●**_	_**○**_
Temporal lobe				
Heschl L	_**○**_	_**○**_	_**●**_	_**○**_
Temporal Sup	_**○**_	_**○**_	_**●**_	_**○**_
Temporal Inf	_**○**_	_**○**_	_**●**_	_**○**_
Parietal lobe				
Parietal Sup	_**○**_	_**○**_	_**○**_	_**●**_
Parietal Inf	_**●**_	_**●**_	_**○**_	_**●**_
SupraMarginal	_**○**_	_**●**_	_**○**_	_**●**_
Angular	_**○**_	_**●**_	_**○**_	_**●**_
Precuneus	_**●**_	_**○**_	_**●**_	_**○**_
Paracental Lobule	_**○**_	_**○**_	_**○**_	_**○**_
Occipital lobe				
Occipital Sup	_**○**_	_**●**_	_**○**_	_**○**_
Occipital Mid	_**○**_	_**○**_	_**○**_	_**●**_
Occipital Inf	_**○**_	_**○**_	_**○**_	_**○**_
Fusiform	_**○**_	_**●**_	_**●**_	_**●**_

The group-wise differences at the whole-brain level were classified by VBM against the healthy elderly group using maximum of the *t*-tests statistics (separated by more than 1 mm) within a cluster and then labelled according to automated anatomical labelling implemented in SPM.

A striking feature of the regions of atrophy shared across subtypes was that all have been identified previously as either members of the rich club^38^ or functional binding nodes,^39^ or as regions having higher than average connectivity. As shown in Supplementary Fig. 3 and in Table 2, the rich club members identified as undergoing atrophy in all four bvFTD subtypes were superior frontal gyrus, thalamus, pallidum, putamen and hippocampus. The functional ‘binding’ regions^39^ common to the four subtypes include anterior cingulate and insula. In addition, parahippocampal gyrus, amygdala and caudate have been identified as highly connected nodes.

By contrast with these subtype-independent regions, the regions listed in Table 3 had more limited subtype overlap and were generally unilateral. Atrophy in the superior temporal gyrus was unique to the TD subtype. The FTP subtype showed atrophy in middle occipital gyrus and precuneus, and the latter is also seen in the FT subtype. There was no overlap between atrophy and any of the rich club or linker regions that was unique to the SL subtype. Degeneration in the superior occipital lobe was unique to the FD subtype. Atrophy in the superior temporal gyrus, although a functional binding node, was unique to the TD subtype.

### Cognitive, functional and behavioural performance across bvFTD subtypes

**Figure 4:**
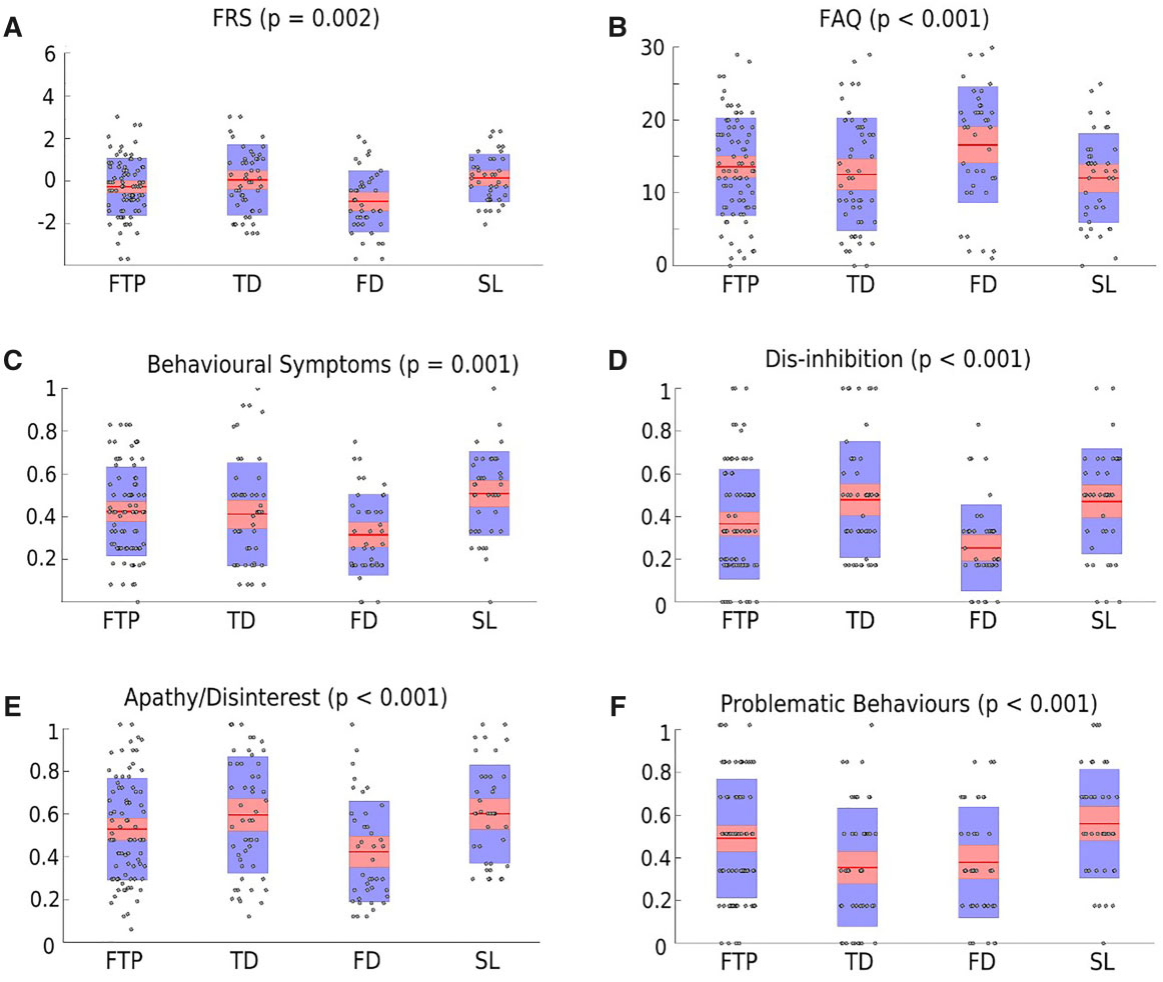
**Behavioural scores by subtype.** (**A**) Frontotemporal Dementia Rating Score; (**B**) Functional Assessment Questionnaire; (**C**) behavioural symptoms; (**D**) disinhibition; (**E**) apathy/disinterest; and (**F**) problematic behaviours. Box-plots with individual data points superimposed for behavioural and functional sub-scores for bvFTD subtypes.

The principal cognitive scales, ACE-R and MMSE, showed no significant differences according to the bvFTD subtypes (Table 4). By contrast, the functional and behavioural scales, FAQ and FRS, showed significant differences (Table 4 and Fig. 4), with the FD subtype showing greater overall impairment than the others. To examine this further, we used FRS subscales to determine whether behavioural elements of the bvFTD syndrome could be differentiated according to subtype. As shown in Table 4 and Fig. 4, a picture like that seen with the full scales emerged, namely that the FD subtype was generally more impaired than the others. This could be seen for behavioural symptoms (characterized predominantly by lack of appropriate behaviours), apathy/disinterest and disinhibition. The only significant exception was that both the FD and TD subtypes were characterized by significantly greater impairment on the subscales measuring problematic behaviours than the FTL and SL subtypes. Although it was possible to map functional and behavioural deficits to specific cortical regions (Supplementary Figs 7 and 8), these features did not discriminate between the bvFTD subtypes identified by structural criteria, apart from greater general impairment largely restricted to the FD subtype (see also Supplementary Fig. 7).

**Table 4:** Behavioural and cognitive variables used in the study in four bvFTD subtypes and corresponding demographic data

Clinical Characteristics	FTP (*N* = 82)	FD (*N* = 41)	SL (*N* = 39)	TD (*N* = 51)	*P*-value
Behavioural and functional					
FRS	−0.45 (1.37)	**−1.10 (1.43)** ^a^ ^,^ ^b^	−0.01 (1.1)	−0.10 (1.65)	0.02
Behavioural symptoms	0.42 (0.21)	**0.31 (0.19)** ^b^ ^,^ ^c^	0.51 (0.19)	0.41 (0.24)	0.001
Cognition	0.36 (0.36)	0.33 (0.39)	0.33 (0.31)	0.43 (0.36)	0.54
ADL	0.52 (0.23)	**0.41 (0.23)** ^a^ ^,^ ^b^	0.59 (0.22)	0.58 (0.27)	0.003
Apathy/disinterest	0.36 (0.26)	**0.25 (0.20)** ^a^ ^,^ ^b^	0.47 (0.24)	**0.48 (0.27)** ^d^	0.001
Eating behaviours	0.63 (0.29)	0.55 (0.27)	0.68 (0.26)	0.54 (0.27)	0.08
Disinhibition	0.42 (0.40)	**0.22 (0.30)** ^b^ ^,^ ^c^	0.56 (0.34)	**0.35 (0.35)** ^e^	0.004
Problem behaviours	0.47 (0.24)	**0.37 (0.25)** ^b^	0.55 (0.24)	**0.35 (0.27)** ^d^ ^,^ ^e^	0.001
FAQ score	13.6 (7.0)	**16.6 (7.8)** ^b^ ^,^ ^d^	12.0 (6.1)	12.5 (7.8)	0.02
UPDRS score	59 (8)	57 (16)	58 (11)	58 (9)	0.56
Cognitive scores					
ACE-R	68 (17)	68 (17)	70 (18)	68 (13)	0.82
Attention	3.8 (1.5)	**3.6 (1.6)** ^b^	3.8 (1.5)	4.5 (1.2)	0.02
Orientation	8.2 (2.0)	7.7 (2.2)	8.3 (2.0)	8.5 (1.7)	0.23
Category fluency	2.8 (2.1)	2.3 (2.0)	3.0 (2.1)	2.6 (1.7)	0.36
Letter fluency	2.8 (2.1)	**2.2 (1.8)** ^a^	2.8 (2.1)	3.4 (1.8)	0.038
Episodic memory	13.0 (5.2)	13.4 (5.4)	12.5 (4.7)	12.6 (4.7)	0.84
Semantic memory	2.5 (1.2)	2.7 (1.2)	2.9 (1.2)	**1.8 (1.3)** ^a^ ^,^ ^d^ ^,^ ^e^	<0.001
Perceptual abilities	**7.1 (1.2)** ^d^	**7.0 (1.0)** ^a^	7.5 (1.3)	7.8 (0.7)	0.006
Praxis	5.0 (2.4)	5.5 (2.1)	5.4 (2.6)	6.0 (2.0)	0.08
Language–phonemics	**1.3 (0.8)** ^c^	1.7 (0.6)	1.6 (0.6)	1.6 (0.7)	0.013
Language–structure	6.2 (1.2)	6.0 (1.1)	6.4 (0.8)	6.1 (1.2)	0.55
Language–semantics	13.8 (3.9)	13.7 (3.6)	14.2 (2.6)	**8.9 (4.7)** ^a^ ^,^ ^d^ ^,^ ^e^	<0.001
MMSE	24.5 (3.7)	23.2 (4.1)	24.5 (4.3)	25.5 (3.5)	0.09
Medication					
AChEI/Mem	64/18	34/7	29/10	44/7	0.48^f^
Demographics					
Age (years)	63.5 (7.5)	63.3 (7.4)	63.4 (7.3)	62.6 (7.8)	0.33
Education (years)	14.6 (6.0)	16.0 (6.0)	15.7 (6.6)	15.0 (6.0)	0.30
Gender (M/F)	53/29	21/20	25/14	37/14	

Significant (*P<*0.05) differences between the bvFTD subtypes in behavioural and cognitive sub-scores are indicated in bold.

a Differences between FD and TD.

b Differences between FD and SL.

c Differences between FD and FTP.

d Differences between TD and FTP.

e Differences between TD and SL.

f *P*-value reported for the Pearson’s Chi-square test.

FTP = frontotemporoparietal; TD = temporal-dominant; FD = frontal-dominant; SL = sub-lobar; FRS = Frontotemporal Dementia Rating Scale; ACE-R = Addenbrooke’s Cognitive Examination Revised; MMSE = Mini-Mental State Examination; FAQ = Functional Assessment Questionnaire; UPDRS = Unified Parkinson’s Disease Rating Scale; AChEI/Mem = acetylcholinesterase inhibitor and/or memantine (1 = taking medication/s); M = male; F = female.

**Figure 5:**
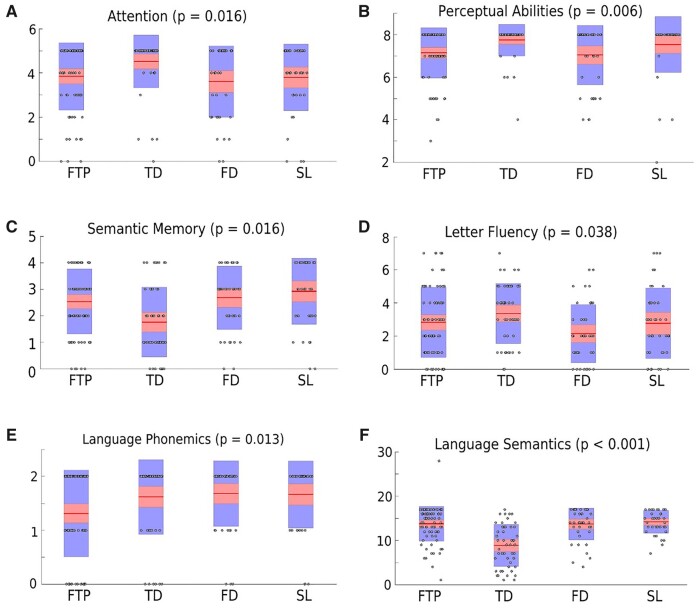
**Cognitive scores by subtype.** (**A**) Attention; (**B**) perceptual abilities; (**C**) semantic memory; (**D**) letter fluency; (**E**) language phonemics; and (**F**) language semantics. Box plots with individual data points superimposed for cognitive sub-scores for bvFTD subtypes.

Since overall cognitive impairment did not provide a basis for discriminating between the subtypes, we next tested whether cognitive subdomains possessed greater discriminatory capacity. Here, a more complex picture emerged, as shown in Table 4, Fig. 5 and Supplementary Fig. 8. As expected, the TD subtype was characterized more specifically by greater impairment in semantic memory and language semantics, but not in episodic memory. The FD subtype was differentiated by more prominent deficits in letter fluency. The FTP subtype showed somewhat greater impairment in language phonemics and perceptual abilities. There were no cognitive deficits that could be linked more specifically to the SL subtype. Although the FTD subtype might appear to be more Alzheimer’s disease-like, there were no differences in likelihood of being prescribed symptomatic treatments approved for Alzheimer’s disease.

### A *post hoc* examination of the relationship between anatomical core features and cognitive, behaviour and functional performance in bvFTD

It is clear from the foregoing analysis that atrophy patterns in bvFTD can be split into those atrophy patterns, which are common to all subtypes and those that are associated with specific subtypes. Using the core regions, we performed a principal components data reduction and found that the first unrotated factor explained 40% of the variance found across all core regions. There were significant correlations between this extracted factor and cognitive, behaviour and functional scores (Table 5). We next used a GLM approach to determine whether these associations depend on unique features of the subtype. In this analysis, the cognitive, behaviour and functional scores were considered separately as the dependent variable, and sex and subtype were included as fixed factors, while age, the summary core factor and head size were included as covariates (Table 5). The GLM analysis showed that the summary core feature drives the association with the cognitive scores and the functional activity score. By contrast, the behavioural score is associated with the cortical subtype, but not with the summary core feature.

**Table 5:** Statistical analysis: correlation between cognitive, behaviour and functional scores and the regional core factor score

Clinical scores	**Core factor**					**Group**		
	*r*	*P*	*F*	*P*	*Post hoc*	*F*	*P*	*Post hoc*
ACE-R	0.44	**<0.001**	22.7	**<0.001**	FTP/FD	1.43	0.24	
FAQ	−0.22	**0.001**	5.04	**0.03**	TD/FD	1.82	0.15	
MMSE	0.34	**<0.001**	9.80	**0.02**	TD/FD	0.51	0.68	
Behaviour	0.14	**0.038**	3.24	0.07		3.19	**0.03**	FD/SL
UPDRS	−0.050	0.47	0.10	0.75		0.69	0.56	

The General Linear Model (GLM) output by core factor and by group is shown for the association between the cognitive, behavioural and functional scores and the regional factor score after adjusting for sex and subtype age and head size.

Pearson correlation (*r*) was determined for the core factor; two-tailed *P*-value; significant values in bold. *Post-hoc* analysis for group differences significant at *P* < 0.05.

ACE-R = Addenbrooke’s Cognitive Examination Revised; FAQ = Functional Assessment Questionnaire; FTP = frontotemporoparietal; FD = frontal-dominant; GLM = general linear model; MMSE = Mini-Mental State Examination; Behaviour = behavioural sub-score from Frontotemporal Dementia Rating Scale; SL = sub-lobar; TD = temporal-dominant; UPDRS = Unified Parkinson’s Disease Rating Scale.

## Discussion

We have analysed a large clinical cohort of 213 well-characterized bvFTD patients from a global, multicentre study. We aimed to examine how common and heterogeneous patterns of atrophy account for clinical diversity in this syndrome. Whilst confirming the existence of four anatomically distinct subtypes at the cortical level, our study has identified SL and limbic brain atrophy as a core feature of bvFTD that is common to the anatomically distinct subtypes that have been described. A summary metric based on core region atrophy was found to associate significantly with cognitive and functional performance across all subtypes. Conversely, cortical heterogeneity was associated with behavioural performance independent of the variance explained by the core features. Therefore, the bvFTD syndrome can be understood as comprising a core disturbance in highly connected subcortical and limbic brain structures that is closely linked to cognitive and functional impairment.

### Common and distinct atrophy patterns

Despite the existence of four anatomically distinct patterns of cortical surface atrophy in the population we have analysed, there is a homogenous pattern of atrophy across subcortical and limbic regions that is common to the anatomical subtypes. Of 39 brain regions showing atrophy when compared to healthy elderly subjects, 16 regions were found to be common to all four subtypes, whereas 23 had selective subtype associations. Of the 16 subtype-independent regions of atrophy, 10 have been characterized previously as brain hubs, i.e. brain regions with higher than average connectivity. These 10 regions map either to the so-called ‘Rich Club’ of highly connected nodes,^38^ to highly connected functional binding nodes^39^ or nodes known to have higher than average connectivity in either functional or structural MRI studies.^40^^,^^41^ These network hubs are central to communication and functional integration of the brain and represent potential hotspots for loss of connectivity across multiple brain networks.^42^ It is known from previous studies that the sub-networks of highly connected nodes play an important role in efficient information processing between segregated brain areas^43^ and have been found to be associated with cognitive performance^44^ in healthy brain. Meta-analysis of MRI studies has suggested that the structural brain hubs and their connections are highly vulnerable to neurodegeneration^45^^,^^46^ although the hubs implicated in bvFTD and Alzheimer’s disease differ.^47^ Of the 23 regions where atrophy is not shared across the subtypes, two were found to be either subcortical or limbic, and five are members of the rich club or functional binding group. We therefore conclude that degeneration in basal, limbic and frontal networks that have high levels of connectivity represents a core feature of the bvFTD syndrome irrespective of the distinct cortical subtypes that have been described.

Degeneration of brain network hubs is not unique to bvFTD. It has been noted that neurodegeneration targets brain hubs in most of the neurodegenerative disorders.^48–50^ As noted above, the anatomical sites of atrophy differ between different neurodegenerative disorders. For example, the central brain regions affected in Alzheimer’s disease are more likely to be the medial temporal and parietal regions, although thalamus and hippocampus are consistently atrophied in both bvFTD and Alzheimer’s disease. It has been proposed that the increased traffic that hubs are required to support may help to explain why these regions have preferentially greater vulnerability to neurological disorders in general.^22^^,^^49^^,^^51^ The vulnerability of the central networks of the human brain to neurodegeneration^49^ may explain the involvement of some rich club network nodes (insula, anterior cingulate, hippocampus, superior temporal pole, pallidum and thalamus), but not all (putamen and a number of cortical regions). A high degree of connectivity may also make certain regions more vulnerable to prion-like spread of pathology arising stochastically in linked subregions. These need not be mutually exclusive, since a chronically high level of activity may itself lead to high demands on turnover of vulnerable protein systems and predispose to pathological protein aggregation and transmission.

The core regions identified in this study underpin brain functions relevant to the core clinical diagnostic symptoms of bvFTD. The superior frontal gyrus is involved in self-awareness, cognitive control, emotion regulation and impulse control. Reduced volumes in FTD have been associated with disinhibition^52^ and ADL dysfunction.^53^ Striatal damage is strongly linked with executive dysfunction, impaired reward–punishment processing, and affective and motivational disturbances.^54^ Striatal atrophy occurs early in bvFTD and has also been associated with eating changes. The von Economo neurons (VENs) of the anterior cingulate cortex, important for empathy, social awareness and self-control, are severely depleted early in bvFTD.^55^ VENs from the anterior insula are thought important for awareness and together form a network with the striatum and amygdala. The amygdala plays a prominent role in mediating decision making, emotional learning and behaviour and is often affected early in bvFTD, particularly in cases due to *MAPT* mutations.^56^ The thalamus is a complex modulatory gate. The anteroventral and dorsomedial nuclei form part of the dorsolateral prefrontal circuit, related to executive function and motor programming, and are also part of the lateral orbitofrontal circuit, related to personality and mood regulation.^57^ Thalamic atrophy is particularly prominent in cases with TDP-43 pathology and C9orf72 mutations.^57^

In an admittedly simplified model, Seeley et al.^58^ have proposed that neurodegeneration in bvFTD targets primarily the salience network. It is hypothesized that this would be responsible for social-emotional-autonomic processing^59^ and affect some other functional brain networks via its afferent/efferent interactions. The salience network is closely allied with the ventral valuation/context appraisal network also known as the semantic appraisal network,^60^ default mode network and task-control or executive network,^61^^,^^62^ all reported as being disrupted in bvFTD. The trans-modal areas of the default mode and salience network also overlap with the rich club network regions.^63^^,^^64^ Our results support the existence of a common underlying pattern of degeneration, which is not restricted to the salience network. Different cortical subtypes of bvFTD ranging from absence of cortical lobar atrophy, to lobe-specific dominance, to multi-lobar atrophy, all share degeneration in the basal, limbic and frontal networks we have described.

In our cohort, the FTP subtype had the highest frequency (39%) and the FD (19%), the TD (24%) and the SL subtypes (18%) had comparable lower frequencies. Therefore, the syndrome as defined by consensus clinical criteria and by the requirement for a significant degree of frontal and/or temporal lobe atrophy remains neuroanatomically heterogeneous in the population we have studied. Our results align with two smaller studies reporting consistent differences in patterns of degeneration across cortical areas in patients diagnosed as having bvFTD by consensus criteria.^11^^,^^13^ This consistency between studies is preserved despite sampling of different subsets of regional volumes and utilization of different statistical classifications. The FD, TD and FTP groups are comparable with the same anatomical subtypes identified by Whitwell et al.^13^ Ranasinghe et al.^11^ designate essentially the same anatomical subtypes in terms of a theoretical construct as the salience-network-frontal subtype (i.e. FD), the semantic appraisal-network subtype (i.e. TD) and salience-network-frontotemporal (i.e. fronto-temporo-parietal) subtype. Their subcortical subtype parallels our SL subtype. Although it is possible that the SL subtype might represent an earlier stage of the disease, Ranasinghe et al. have argued that it represents a true bvFTD subtype, which progresses more slowly. We found no global cognitive, functional or behaviour differences, which might have been expected if it represented an earlier stage of the disease. Our data therefore support the suggestion of Ranasinghe et al. that this is indeed a distinct subtype, and that its prevalence is comparable to that of the FD and TD subtypes.

Heterogeneity at the cortical level is associated to only a limited extent with distinct behavioural, functional and cognitive features. The FD subtype is characterized by greater global impairment on both the FRS and FAQ scales. It is notable that the FD subtype is the most severely affected in terms of behavioural symptoms, such as lack of appropriate social response, apathy and disinterest, as well as disinhibition and problematic positive behaviours. In other words, in contrast to the overall importance of highly connected networks in defining the bvFTD syndrome, the frontal lobe remains particularly important for regulation of behaviour. By contrast, cognitive deficits segregate as expected, with the TD form associated particularly with semantic memory and language semantics, and a stronger association between FD atrophy and impairment in letter fluency.

Independently of the subtype, a summary metric variable for the core features (the first unrotated factor), was found to have a highly significant statistical association with cognitive impairment, particularly with ACE-R, and with functional impairment as measured by the FAQ scale. After adjusting for the core factor, there was no residual association with subtype. On the other hand, the behavioural subscale derived from the FRS retained a significant association with anatomical subtype after taking account of the core factor variable. This may explain the possible latent nature of the pure SL subtype^11^ in which prominent behavioural deficits may not be demonstrated.

This mapping of imaging features to clinical features should be viewed in the context of the inclusion criteria of this study, namely the requirement for presence of brain atrophy in frontal and/or temporal lobes scoring two or more on a scale proposed by Kipps.^25^ That is, subjects with little or no frontal and/or temporal atrophy and who fulfilled all other criteria for bvFTD were not included. The results of the present analysis suggest that the diagnostic utility of MRI in the differentiation of bvFTD from healthy controls and other dementias may be best served by examining the core features with or without the frontal and temporal lobes. A recent study reports that a data driven approach for discriminating between bvFTD patients and controls showed good discriminatory performance without a priori knowledge of any potential structure within the data.^65^ It remains to be determined how a prior knowledge of the core features, we have described could improve the discrimination. More importantly, it remains to be determined how incorporating a core feature metric assists with the more pertinent clinical question, which is to discriminate between bvFTD and other dementias.

### Limitations and conclusions

There are limitations to the inferences which can be drawn from the present study. Although it is based on a large sample, it is possible that still greater power is required to define the subtle clinical features of the subtypes. Similarly, the clinical scales we have used to interrogate the bvFTD phenotype may not be sufficiently discriminatory, and more refined neuropsychological measures may characterize the clinical features of the subtypes with greater subtlety. A further limitation is that we have used patterns of atrophy on MRI as the sole investigative tool for analysing the brain abnormalities of bvFTD. Although this has the advantages of a wide applicability and standardization, metrics based on functional MRI that may be able to define abnormalities in the underlying connectome were not available in this study. Again, there is a trade-off between the feasibility of more refined approaches and study size/cost considerations.

Notwithstanding these limitations, a useful general picture that emerges from our study is that the MRI abnormalities in the bvFTD syndrome can be characterized at two neuroanatomical levels. The core of the syndrome appears to depend on a common pattern of degeneration in which basal and limbic lobes are disproportionately represented. Some, but not all of these, have been identified previously as brain structural and functional network hubs. In addition, the neuroanatomical heterogeneity at the cortical level, which is robust and reproducible across studies, appears to have limited explanatory power in accounting for cognitive, functional and behavioural heterogeneity. Our results are consistent with the idea that bvFTD is characterized by a core disturbance within basal, limbic and frontal networks required for integration of cognition, function and behaviour. This core disturbance at the level of integration may help to understand both the inappropriate conduct that families find distressing and the higher rates of socially inappropriate behaviour and criminality in bvFTD than in other comparable neurodegenerative disorders.^4^ There appears to be a dissociation between the cognitive understanding of actions and their consequences as matters of fact, and the capacity for an appropriate evaluation of their personal and societal implications.^5^^,^^6^ The loss of insight that characterizes the condition could be understood as resulting from pathology affecting particularly the central integrative systems that enable segregated functional regions of the brain to interact and communicate.

## Supplementary material

Supplementary material is available at *Brain Communications* online.

## Supplementary Material

fcab241_Supplementary_Data

## Abbreviations

ABC36 =: Aberdeen 1936 Birth Cohort
ACE-R =: Addenbrooke’s Cognitive Examination Revised
AD =: Alzheimer’s disease
ADL =: activities of daily living
bvFTD =: behavioural variant of frontotemporal dementia
eTIV =: estimated total intracranial volume
FAQ =: Functional Assessment Questionnaire
FD =: frontal-dominant
FRS =: Frontotemporal Dementia Rating Scale
FTD =: frontotemporal dementia
FTP =: frontotemporoparietal
FP =: frontoparietal
GLM =: general linear model
MMSE =: Mini-Mental State Examination
MNI =: Montreal Neurological Institute
ROI =: region of interest
SL =: sub-lobar
TD =: temporal-dominant
VBM =: voxel-based morphometry
