# Supplementary material for: Degeneration of basal and limbic networks is a core feature of behavioural variant frontotemporal dementia
Source: Brain Commun. 2021 Oct 21;3(4):fcab241. doi: 10.1093/braincomms/fcab241 (PMC8688778; doi:10.1093/braincomms/fcab241)
Supplement: fcab241_Supplementary_Data [file fcab241_supplementary_data.docx]

**Supplementary Information**

**Degeneration of basal and limbic networks is a core feature of behavioural variant frontotemporal dementia**

Vesna Vuksanović,^1,2,4^ Roger T. Staff,^3^ Suzannah Morson,^4,5^ Trevor Ahearn,^3^ Luc Bracoud,^6^ Alison D. Murray,^2^ Peter Bentham,^4^ Christopher M. Kipps,^7^ Charles R. Harrington^2,4^ and Claude M. Wischik^2,4^

^1^ Swansea University Medical School, Health Data Research UK, Swansea University, Swansea, UK.

^2^School of Medicine, Medical Sciences and Nutrition, University of Aberdeen, Aberdeen, UK

^3^Medical Physics, NHS Grampian, Aberdeen, UK

^4^TauRx Therapeutics, Aberdeen, UK

^5^School of Psychology, University of Aberdeen, Aberdeen, UK

^6^Bioclinica, Lyon, France

^7^University Hospital Southampton and University of Southampton, Southampton, UK

# Clinical Assessment Tools

Assessments of the severity of impairment, whether cognitive or behavioural, consist of a fixed set of items that are administered then summed to give an overall severity (or ability) score. This score can then be compared to established norms to identify whether a subject is within the range of acceptable performance or whether an impairment has been identified. While this is informative for diagnostic purposes, reliance on the overall score removes an important information. For example, take two subjects of the same demographic, Subject A and Subject B. Both subjects score 75/100 on the ACE-R, a score that would identify them as cognitively impaired. However, by looking at the specific items that each subject was unable to correctly answer, we can see that Subject A predominantly had difficulty with language-based items, while Subject B predominantly had difficulty with memory-based items. By looking at the finer grained information, we are able to say more about our subjects than the simple observation that both have a cognitive impairment.

There are many assessments where subscales are already identified and used by clinicians and researchers alike. The ACE-R, for example, identifies five such subscales or domains: attention and orientation, memory, verbal fluency, language, and visuo-spatial ability. Although no explicit subscales are provided, the FRS is also widely acknowledged to contain items to measure both behavioural symptoms and functional abilities. While more informative than their total score counterparts, it is still possible to obtain even finer grained information from these assessments than those subscales identified above. For example, the verbal fluency subscale from the ACE-R contains measures of both letter and category fluency, which may be disproportionately impaired dependent on the location of brain atrophy (Possin *et al.,* 2013).

Items from the ACE-R and the FRS were discussed by a psychiatrist (PB) and a psychologist (SM) until a consensus was reached. A total of 18 derived measures were identified: 11 from the ACE-R and 7 from the FRS.

**1.1. Sub-scores Derived from ACE-R**

The Orientation and attention domain from the ACE-R was divided into two separate measures of orientation and of attention. Orientation items primarily examine orientation to time and place, while attention includes mental arithmetic and 3-word repetition. Three-word repetition was included as an attention measure as it was considered to be too short a period to engage episodic memory processes. Verbal fluency was similarly divided to give a letter fluency measure and a category fluency measure. Episodic memory and semantic memory measures were obtained from the Memory domain. Episodic memory items were those for which recall was tested of materials encountered within the confines of the test, for example name and address recall. Semantic memory relies on knowledge learned outside of the confines of the test that is often well rehearsed, whether explicitly or implicitly. Semantic memory items were those that do not draw on personal experience, but are considered to be general knowledge, for example the name of the current President of the USA. Language items were split to give 3 separate language measures: phonemics, semantics and structure. Language-phonemics reflects those items that required repetition of a single word only, the simplest level of language ability. Language-semantics involves items that require some knowledge of the meaning behind individual words to be able to successfully respond to the item in question. Language-structure reflects those items that the full grammatical structure of a sentence is needed to allow the subject to respond to the item correctly. Finally, the visuo-spatial domain was split into two separate measures of praxis (motor planning) and perceptual abilities.

The items contained within the eleven subscores based on the ACE-R are as follows:

**Orientation (Score 0-10)**

- Ask what is the… day/ date/ month/ year/ season
- Ask which… building/ floor/ town/ county/ country

**Attention (Score 0-8)**

- I’m going to give you three words and I would like you to repeat after me…
- Could you take 7 away from 100?...

**Letter fluency (Score 0-7)**

- I’m going to give you a letter of the alphabet and I’d like you to say as many words as you can beginning with that letter…

**Category fluency (Score 0-7)**

- Now can you name as many animals as possible, beginning with any letter

**Episodic memory (Score 0-22)**

- Which 3 words did I ask you to repeat and remember?
- I’m going to give you a name and address and I’d like you to repeat after me…
- Now tell me what you remember of that name and address we were repeating at the beginning
- Address recognition: ok, I’ll give you some hints, was it…

**Semantic memory (Score 0-4)**

- Name of the current US president
- Name of the person who was president before him/her
- Name of the current Queen or King of England
- Name of the British Prime Minister during WWII

**Language – phonemics (Score 0-2)**

- Ask the subject to repeat: hippopotamus, eccentricity, unintelligible, statistician

**Language – semantics (Score 0-17)**

- Ask the subject to name the following pictures…
- Using the pictures above, ask the subject to…
- Ask the subject to read the following words

**Language- structure (Score 0-7)**

- Show written instruction: Close your eyes
- Take the paper in your right hand. Fold the paper in half. Put the paper on the floor.
- Ask the subject to make up a sentence and write it in the space below
- Ask the subject to repeat: Above, beyond and below; No ifs ands or buts

**Praxis (Score 0-8)**

- Ask the subject to copy this diagram: Overlapping pentagons
- Ask the subject to copy this drawing: Wire cube
- Ask the subject to draw a clock face with numbers and the hands at ten past five

**Perceptual (Score 0-8)**

- Ask the subject to count the dots without pointing to them
- Ask the subject to identify the letters

The items contained within the seven subscores obtained from the FRS are as follows:

**Cognition (Score 0-2)**

- Becomes confused or muddled in unusual surroundings
- Forgets what day it is

**Behavioural (Score 0-12)**

- Lacks interest in doing things: their own interests/leisure activities/new things
- Lacks normal affection, lacks interest in family members worries
- Is uncooperative when asked to do something; refuses help
- Is restless
- Acts impulsively without thinking, lacks judgement
- Lacks interest or motivation to perform household chores that he/she used to perform in the past
- Lacks interest in his/her personal affairs such as finances
- Lacks previous interest or motivation to prepare a meal (or breakfast, sandwich) for himself/herself
- Lacks initiative to eat
- Has problems eating meals at a normal pace and with appropriate manners
- Wants to eat the same foods repeatedly
- Prefers sweet foods more than before

**Activities of daily living (Score 0-17)**

- Has problems taking his/her usual transportation safely
- Has difficulties shopping on their own
- Has difficulties completing household shores adequately that he/she used to perform in the past (to the same level)
- Has difficulty finding and dialling a telephone number correctly
- Has problems organising his/her finances and to pay bills
- Has difficulties organising his/her correspondence without help
- Has problems handling adequately cash in shops, petrol stations, etc.
- Has problems taking his/her medication at the correct time
- Has problems taking his/her medication as prescribed
- Has difficulties organising the preparation of meals (or a snack if patient was not the main cook)
- Has problems preparing or cooking a meal (or snack if applicable) on their own
- Has difficulties choosing appropriate utensils and seasonings when eating
- Has problems eating meals at a normal pace and with appropriate manners
- Has problems choosing appropriate clothing
- Is incontinent
- Cannot be left at home by himself/herself for a whole day
- Is restricted to the bed

**Apathy/disinterest (Score 0-6)**

- Lacks interest in doing things: their own interests/leisure activities/new things
- Lacks normal affection, lacks interest in family members worries
- Lacks interest or motivation to perform household chores that he/she used to perform in the past
- Lacks interest in his/her personal affairs such as finances
- Lacks previous interest or motivation to prepare a meal (or breakfast, sandwich) for himself/herself
- Lacks initiative to eat

**Abnormal eating behaviour (Score 0-4)**

- Lacks initiative to eat
- Has problems eating meals at a normal pace and with appropriate manners
- Wants to eat the same food repeatedly
- Prefers sweet foods more

**Positive problem behaviour (Score 0-6)**

- Is uncooperative when asked to do something; refuses help
- Is restless
- Acts impulsively without thinking, lacks judgement
- Has problems eating meals at a normal pace and with appropriate manners
- Wants to eat the same foods repeatedly
- Prefers sweet foods more than before

**Disinhibition (Score 0-2)**

- Acts impulsively without thinking, lacks judgement
- Has problems eating meals at a normal pace and with appropriate manners

**1.2. Sub-scores Derived from FRS**

The FRS presents items grouped into categories relating to the objective area of activity affected, e.g. finance, medication and meals and eating behaviour. While useful for maintaining test cohesiveness and face validity, these categories are not as useful clinically to identify the precise behavioural problems occurring. For example, while it may be informative for the patient and carer to know that finances are a particular struggle, it is more informative clinically to note that several items reflecting apathy or lack of interest are being observed. Subsequently, seven measures reflecting clinically useful symptoms were identified. First, items assessing primarily cognitive ability were assigned as a cognitive measure. Second, a general behaviour measure was identified, covering all items that specifically looked at behavioural symptoms. The third measure was activities of daily living (ADL), reflecting items that closely resembled those of ADL specific scales. Apathy/disinterest was also identified as a measure, regardless of the category in which it occurred (e.g. household chores, finances). Abnormal eating behaviour was identified as a measure, separating it from meal preparation. The positive problem behaviours measure acts as the antithesis of the apathy measure, encompassing those behaviours that the presence of which creates problems for the patient or the carers. The final measure is disinhibition, behaviours showing a lack of restraint or abidance to social norms. While the ACE-R can be divided into distinct measures, items from the FRS may contribute to one or more measure. For example, the item lacks initiative to eat can be categorised as an exhibition of apathy but is also an example of abnormal eating behaviour.

1. **Neuroimaging Data and Classification of bvFTD Patients**

Clustering analysis was performed on 68 regions from the Desikan-Killiany Atlas (DKA) parcellation of the cortex (Desikan *et al.,* 2006). Both left and right hemispheres were considered from the following regions:

Amygdala

Caudal anterior cingulate

Caudal middle frontal

Caudate

Entorhinal

Frontal pole

Fusiform

Hippocampus

Inferior parietal

Inferior temporal

Insula

Isthmus of cingulate

Lateral orbitofrontal

Medial orbitofrontal

Middle temporal

Pallidum

Paracentral

Parahippocampal

Pars opercularis

Pars orbitalis

Pars triangularis

Pericalcarine

Post central

Posterior cingulate

Precentral

Putamen

Rostral anterior cingulate

Rostral middle frontal

Superior frontal

Superior parietal

Superior temporal

Temporal pole

Thalamus

Transverse temporal

- 1. **Dendrogram**


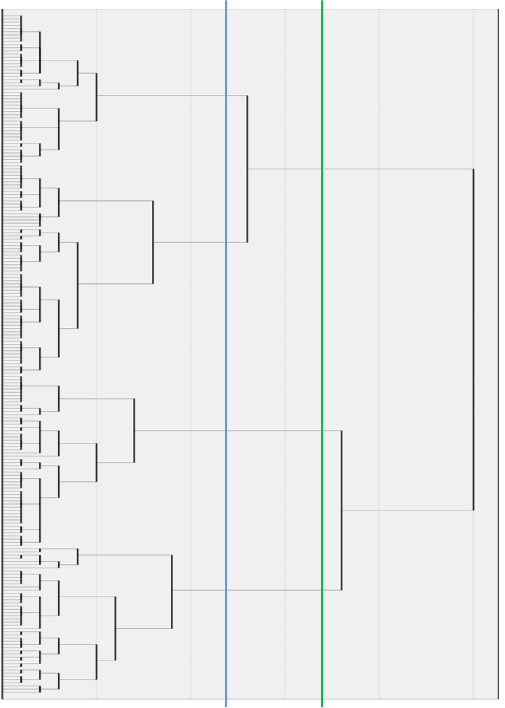


**Supplementary Fig. 1. The dendrogram created by the hierarchical agglomerative clustering.** Two hundred and thirteen behavioural variant Frontotemporal dementia (bvFTD) individals are progressively linked together using Ward’s method. Vertical lines indicate cut off at either four (blue line) or three (green line) clusters for which we performed voxel-wise comparisons of MR images between the bvFTD and the healthy control group.

- 1. **Comparisons of the four bvFTD sub-groups using Voxel-Based-Morphometry**

The four bvFTD clusters compared with the healthy elderly group using a VBM model that includes correction for estimated total intracranial volumes (Supplementary Fig. 2) and correction for total brain volume (Supplementary Fig. 4). Brain view showing common and group-specific regional differences (Supplementary Fig. 3).


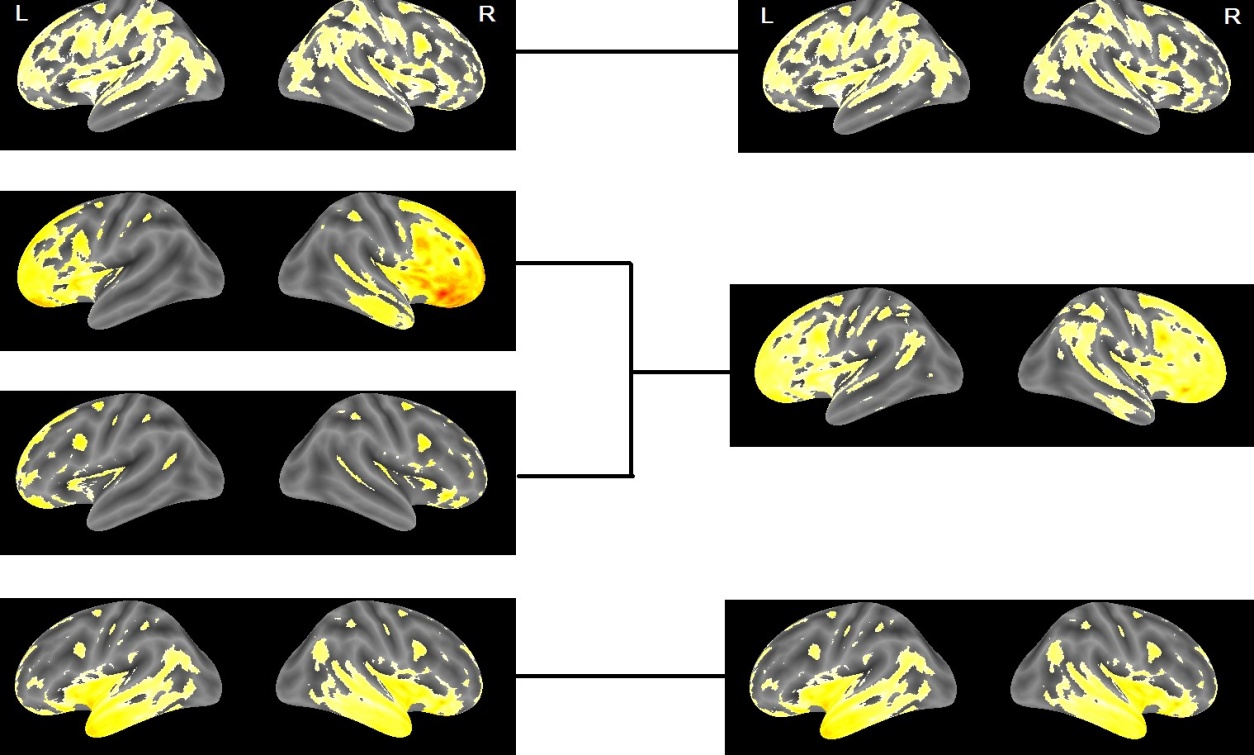


**Supplementary Fig. 2.** **Surface maps for four and three clusters.** 3D surface rendering of differences between healthy controls and each behavioural variant Frontotemporal dementia (bvFTD) subtype (hot areas) visualised at the level of dendrogram when subjects are divided into either four or three groups (denoted by either a blue or a green line respectively in the dendrogram; Fig. 1 of the main text). VBM model used here accounted for differences in estimated intra-cranial volumes between the subjects.


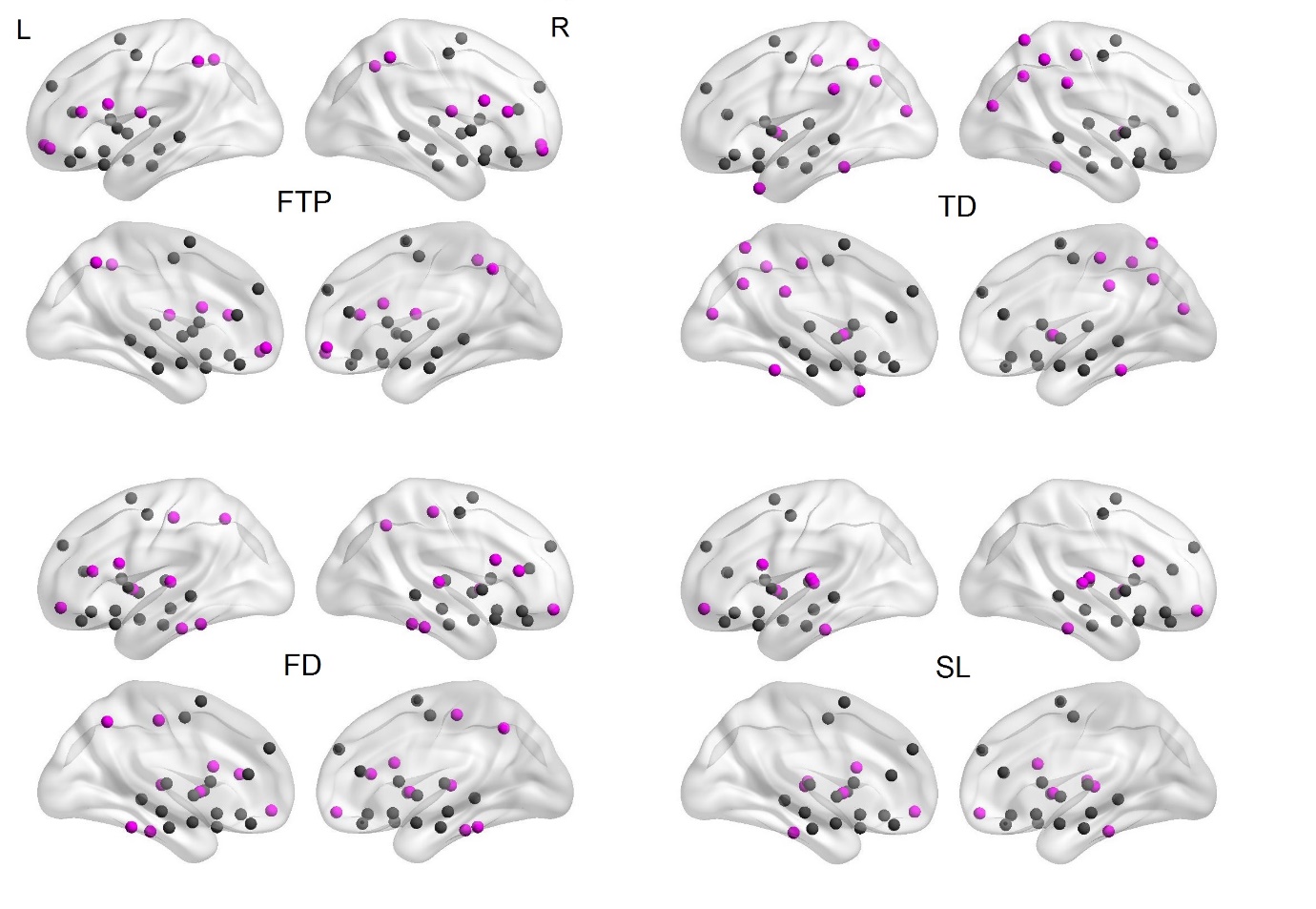


**Supplementary Fig. 3.** **Core and sub-type-specific regions.** Brain views (sagittal and middle) of the common-to-all (grey) and distinct (magenta) regional atrophy in the four bvFTD sub-groups. Atrophied regions were labelled using Automated Anatomical Labelling (AAL). Abbreviations: FTP = frontotemporoparietal; TD = temporal-dominant; FD = frontal-dominant; SL = sub-lobar.


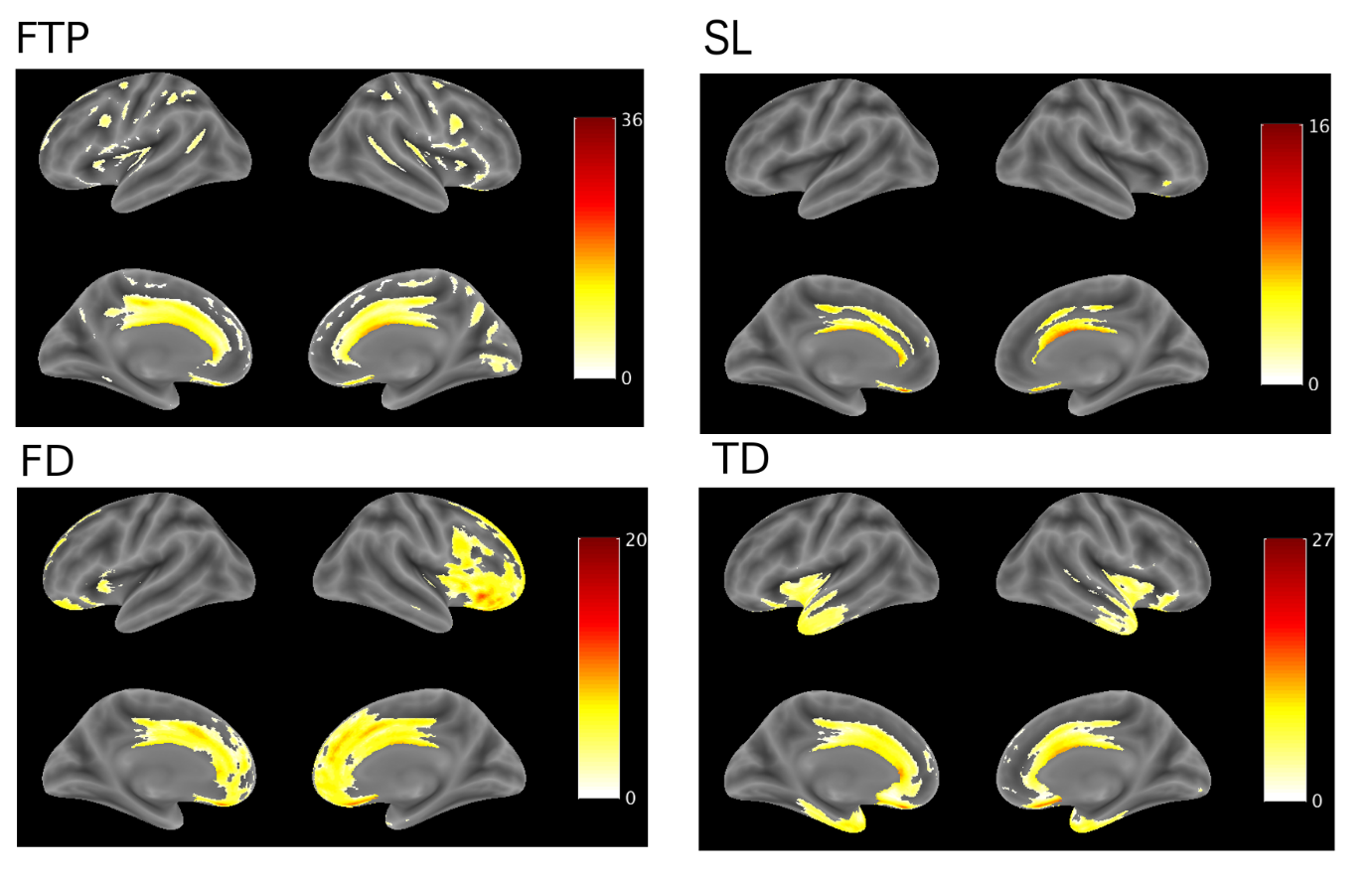


**Supplementary Fig. 4.** **Surface maps for differences between four sub-types.** 3D surface rendering showing patterns of grey matter atrophy of bvFTD clusters in comparison with the healthy controls when corrected for total brain volume.

# 4. Factor reduction and bvFTD subtypes using Principal Component Analysis

**4.1. Factor reduction in grey matter differences**

Supplementary Fig. 5 represents a 3D-scatter plot of the first three components estimated using PCA to explain variability in differences between the subtypes in cortical atrophy. The plot shows good separation of the subtypes in the 3D-space. Factor reduction was calculated in a way that the PCA scores were extracted from the volumes of each of the 68 ROIs used in the cluster analysis. This was done in order to reduce data dimensionality and to understand how much of the total variability in the grey matter differences can be explained using a reduced number of factors. The analysis represents a validation of the clustering approach explained in the main text. The PCA showed that 17 factors, each representing linear combination of the original variables, can explain about 73 % of the total variance in grey matter differences. The first three PCA scores, shown in the plot, explain about 35 % of the total variance. The four subtypes project onto visually distinct areas in the 3D-reduced-factor-space.


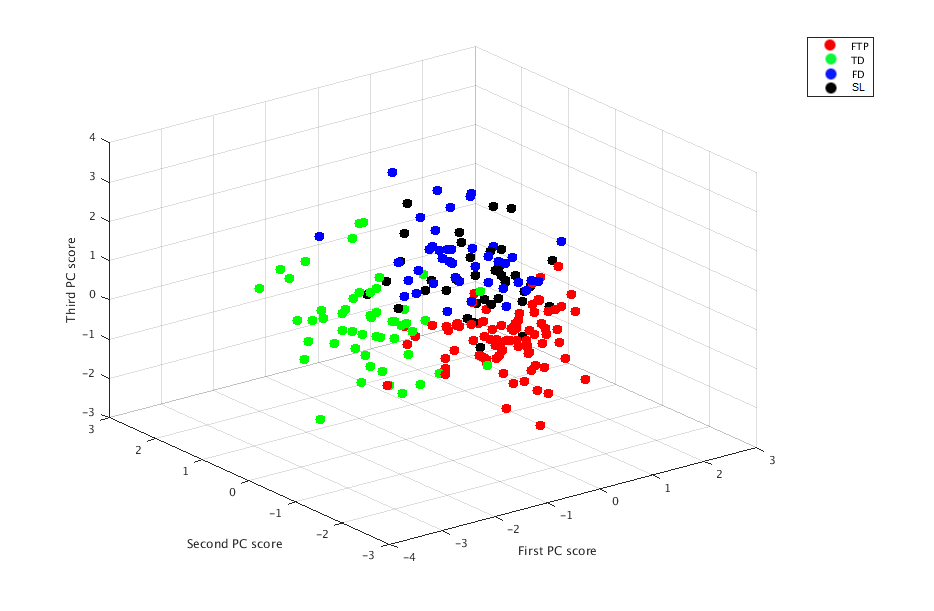


**Supplementary Fig. 5. Principal Component Analysis (PCA) based on 68 cortical regions of interests.** 3D scatter plot showing the separation of the four bvFTD subtypes using the first three components (PCA scores) in the factor reduction analysis on the 68 regions of interest used in hierarchical agglomerative clustering. The first three components explain 35 % of the total variance in the grey matter differences between the subjects. Each of the subtypes occupies a specific area in the reduced factors’ space. Abbreviations: frontal-dominant (FD), frontotemporoparietal (FTP), temporal-dominant (TD) and sub-lobar (SL).

**4.2. Factor reduction in clinical sub-scores**

We also performed PCA to estimate the number of hidden factors within the cognitive and behavioural sub-scores. We wanted to test if the reduced number of factors underlying variability in behavioural and cognitive impairment would also map the differences in the subtypes. We found that five components explain about 70 % of variance in the data. Supplementary Fig. 6 shows the result for the first three components which explain as much as 53.8 % of the total variance in behavioural and cognitive sub-scores. As expected, bvFTD subtypes do not project onto distinct planes in the reduced cognitive-behavioural space.


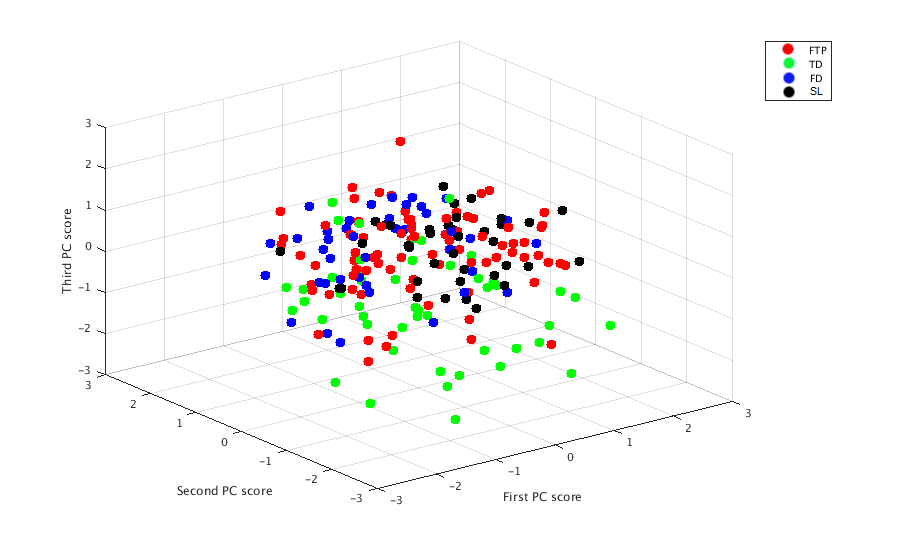


**Supplementary Fig. 6. Principal Component Analysis (PCA) of clinical scores.** 3D scatter plot showing separation of the four bvFTD sub-types using the first three components (PCA scores) in the factor reduction analysis on the cognitive and behavioural sub-scores. The first three components explain 55.4 % of the total variance in cognitive and behavioural outcomes between the subjects. However, there is no clear separation between anatomical sub-types in the reduced space. Abbreviations: frontal-dominant (FD), frontotemporoparietal (FTP), temporal-dominant (TD) and sub-lobar (SL).

**5. Neuroimaging correlates of cognitive, behavioural and functional scores**

Neuroimaging correlates of cognitive, behavioural and functional sub-scores that show significant differences between the four sub-types were calculated on 213 bvFTD subjects (as a single group) using regression analysis implemented in VBM. In the implemented model each voxel’s value was corrected for age and gender and estimated total intracranial volume. An additional variable (a derived sub-scores) was then added to each model to obtain corresponding maps onto the cortical surface areas, as shown in Supplementary Figs. 7 and 8.


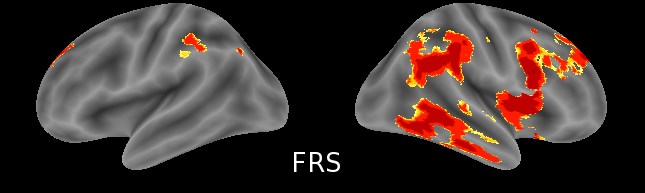

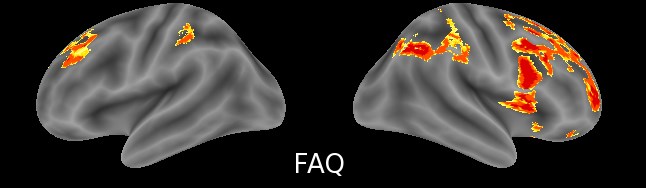

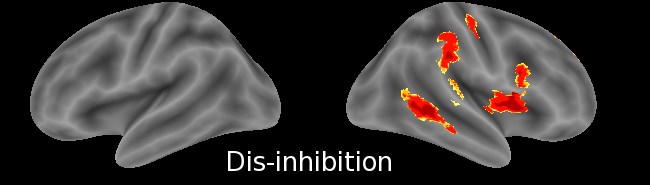

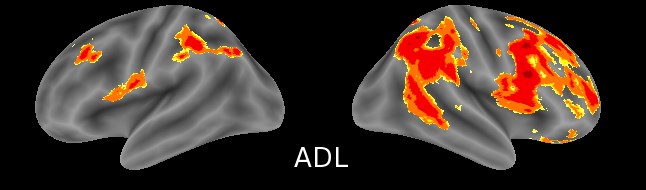

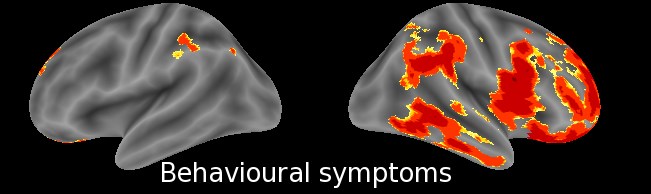

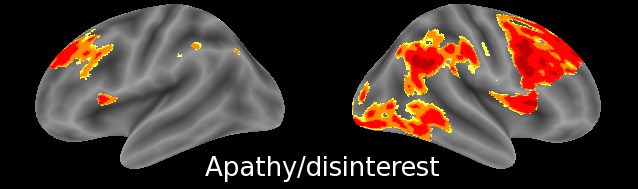

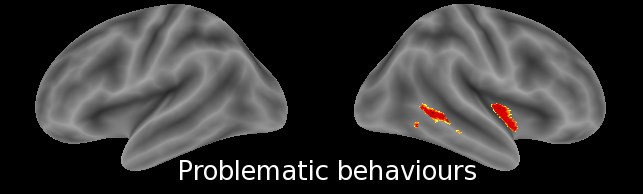

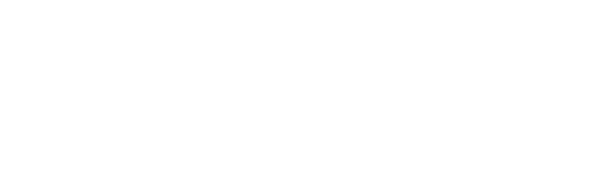


**Supplementary Fig. 7.** **Surface maps for behavioural domains.**  3D surface rendering showing neural correlates of behavioural/functional sub-scores in our group of bvFTD subjects. For the comparisons with bvFTD subtypes see Table 4 and Fig. 4 in the main text. Abbreviations: Frontotemporal Dementia Rating Scale (FRS), Functional Activities Questionnaire (FAQ), Activities of Daily Living (ADL).


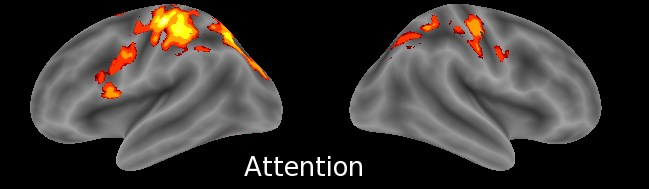

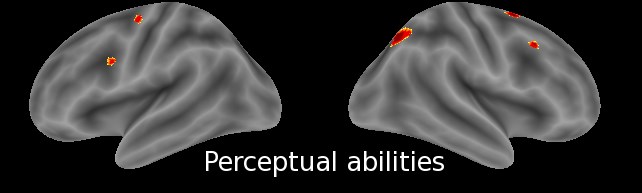

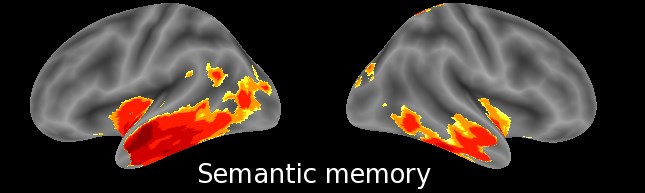

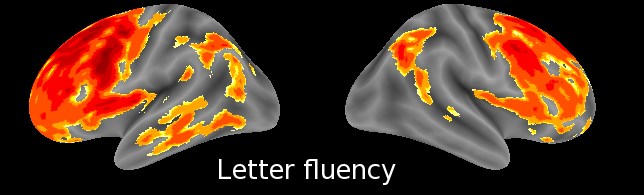

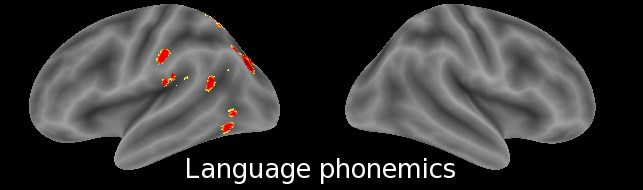

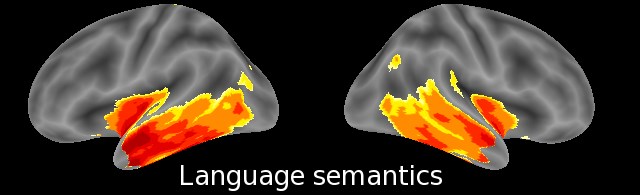


**Supplementary Fig. 8. Surface maps for cognitive domains.** 3D surface rendering showing neural correlates of cognitive sub-scores in our group of bvFTD patients. For the comparisons with bvFTD sub-types see Table 4 and Fig. 5 in the main text.

**References**

Desikan, RS, Ségonne, F, Fischl, B, Quinn, BT, Dickerson, BC, Blacker, D, et al. An automated labeling system for subdividing the human cerebral cortex on MRI scans into gyral based regions of interest. NeuroImage 2006; 31: 968-80.

Possin, KL, Feigenbaum, D, Rankin, KP, Smith, GE, Boxer, AL, Wood, K, et al. Dissociable executive functions in behavioral variant frontotemporal and Alzheimer dementias. Neurology 2013; 80: 2180-85.
